# Targeting Spinal Dorsal Horn Sensitisation in Chronic Pain: Mechanisms and Translational Strategies

**DOI:** 10.1007/s12264-026-01642-1

**Published:** 2026-06-09

**Authors:** Volodymyr Krotov, Olga Kopach

**Affiliations:** 1 Bogomoletz Institute of Physiology, Kyiv, 01024 Ukraine; 2 City St. George’s University of London, London, SW17 0RE UK; 3 Queen Square Institute of Neurology, University College London, London, WC1N 3BG UK

**Keywords:** Chronic pain, Central sensitisation, Spinal cord, Dorsal horn neurons, Nociceptive transmission, Synaptic excitation and inhibition, Maladaptive plasticity, Gene therapy

## Abstract

Chronic pain is a multidimensional, highly debilitating disease arising from diverse functional and structural impairments across the nervous system. Despite heterogeneous aetiologies, chronic pain states converge on shared pathophysiological mechanisms: peripheral and central sensitisation. These dysfunctions reflect long-term functional and structural plasticity within the peripheral and central nociceptive pathways, resulting in amplified signal transmission and pain chronification. Historically, research and drug development prioritised peripheral targets to suppress nociceptive inputs, often neglecting central sensitisation, a core mechanism of treatment-resistant pain. Sensitisation of dorsal horn (DH) circuits underlies network hyperexcitability associated with disrupted excitation-inhibition balance, synaptic potentiation, and circuit reorganisation. This review summarises recent advances in delineating synaptic, cellular, and DH network-level adaptations underlying chronic pain. We discuss emerging mechanism-based strategies to restore circuit balance by targeting cell-type-specific maladaptive plasticity rather than globally suppressing neural activity. Targeted approaches hold promise for improving the specificity, efficacy, and safety of next-generation chronic pain therapies.

## Introduction

Chronic pain is a major global health challenge and a heavy burden for society. It affects approximately 20% of the European population [1], with estimated annual costs of over €200 billion [2]. In the United States, at least 1 in 5 adults experiences chronic pain [3], resulting in an annual economic burden greater than the combined costs of cancer, cardiovascular disease, and diabetes [4]. In the United Kingdom, 35%–51% of individuals report chronic pain, and 10%–14% live with moderate-to-severe pain [5]. Beyond its economic consequences, chronic pain markedly reduced quality of life by restricting professional and social functioning and family life [6–8]. Furthermore, it commonly co-occurs with comorbidities, such as depression, anxiety, substance abuse, sleep disturbances, fatigue, cognitive impairments, and broader mental dysfunction [9–11]. Despite pain prevalence and impact, therapeutic options remain limited: pharmacological relief is often partial, particularly in neuropathic pain, and it is achieved in only 30%–40% of patients [12]. Although chronic pain is now recognised as a disease in its own right [13, 14], clinical management largely prioritises symptomatic relief over targeting underlying pathological mechanisms.

Mechanistically, chronic pain arises from diverse molecular and cellular alterations that vary by aetiology. However, across conditions, it consistently features peripheral and central sensitisation [15, 16], reflecting maladaptive functional, molecular, and structural changes within the peripheral (PNS) and central nervous systems (CNS) that amplify nociceptive signalling and promote pain persistence. Historically, pain research and clinical management have targeted the PNS, primarily because of more accessible targets and therapeutic development not constrained by the blood-brain barrier (BBB) penetration. These approaches include pharmacological and surgical interventions, or both, aimed at suppressing peripheral nociceptor activity and signal transfer. In contrast, CNS-directed therapies remain constrained by modest efficacy and substantial adverse effects. For instance, gabapentinoids and tricyclic antidepressants — first-line treatments for neuropathic pain — commonly cause sedation and dizziness, while opioids are associated with tolerance, dependence, respiratory depression, and even opioid-induced hyperalgesia. Recent advances in drug delivery and genetic approaches that allow cell-type-selective manipulation of central neurons, along with a better understanding of central pain mechanisms, offer new opportunities for mechanism-based interventions targeting the CNS that could lead to the development of novel, more effective therapies for managing pharmacologically refractory pain.

Among the central nociceptive pathways, the spinal dorsal horn (DH) plays a pivotal role in integrating peripheral sensory inputs and pain processing, shaping ascending nociceptive transmission. Neurons in DH laminae I–III receive direct input from high-threshold nociceptive and low-threshold mechanosensory primary afferents, integrate these signals through local microcircuits, and relay processed information to supraspinal centres [17–19]. The DH represents a promising target for CNS-directed therapies for several reasons. Firstly, metabolic and structural alterations associated with chronic pain are localised predominantly within the DH rather than peripheral nerves or dorsal root ganglia (DRG) [20]. Secondly, spinal level interventions can modulate chronic pain of both peripheral and central origins. Thirdly, targeting central sensitisation within the DH would prevent its propagation to supraspinal structures, thereby attenuating aberrant brain pathological processing and reducing pain-related comorbidities.

This review summarises recent advances in understanding neuron-type-specific mechanisms underlying DH sensitisation, with a particular emphasis on works from the past decade. Studies addressing sensitisation mechanisms at the levels of the PNS, supraspinal centres, or descending modulatory pathways are not reviewed to maintain a focused discussion on intrinsic DH microcircuits in chronic pain.

## The Concept of DH Sensitisation and Its Cell-Type-Specificity

Central sensitisation is a core mechanism of chronic pain that encompasses a broad range of cellular and molecular adaptations within the CNS. It is commonly defined as an activity-dependent form of neuronal plasticity that enhances ‘the function of neurons and circuits in nociceptive pathways caused by increases in membrane excitability and synaptic efficacy as well as reduced inhibition’ [16]. In this context, an important unresolved question remains whether DH sensitisation primarily reflects network-level reorganisation—through altered synaptic connectivity and circuit dynamics—or arises mainly from intrinsic hyperexcitability of individual spinal neurons.

A prevailing concept is that DH sensitisation is driven by disrupted excitation and inhibition (E/I) balance; however, the relative contribution of intrinsic cellular versus synaptic mechanisms of neuronal hyperexcitability remains debated. Numerous studies report minimal or no change in passive and active membrane properties of DH neurons in inflammatory [21] and neuropathic [22–26] pain models, whereas others describe increased membrane excitability after spinal cord injury (SCI) [27, 28] or chronic constriction injury (CCI) [29], frequently linked to ion-channel remodelling. In those contexts, hyperexcitability has been associated with upregulation of voltage-gated ion channels, including increased Nav1.3-dependent persistent sodium currents [30], leak sodium channel (NALCN) conductance in neuropathic [31] and inflammatory [32] pain models, and altered L- and N-type calcium channels in diabetic neuropathy [33], contributing to sustained depolarisation. For example, Cav1.2 (L-type) upregulation in wide-dynamic-range (WDR) neurons contributes to neuronal hyperexcitability and mechanical allodynia after spinal nerve ligation [34].

Pain-associated intrinsic plasticity is not uniform across DH neuronal populations. Some cell types show reduced excitability [35], e.g., inhibitory interneurons [36, 37], and both excitatory and inhibitory neurons can exhibit membrane hyperpolarisation [38, 39]. Alterations in membrane excitability may be biphasic, with an initial increase that diminishes over time and ultimately transitions into a decrease, potentially reflecting a homeostatic adaptation to sustained synaptic drive [40]. These indicate that intrinsic changes—when observed—are cell-type-dependent and unlikely to constitute a sole or dominant mechanism of DH sensitisation.

In contrast, potentiation of glutamatergic synaptic excitation is a robust, consistent, and reproducible feature across diverse persistent pain states [22, 23, 41, 42]. It arises from both enhanced primary afferent drive and strengthened excitation within the intrinsic DH network and is increasingly viewed as a dominant driver of DH hyperexcitability. Current evidence indicates that DH sensitisation emerges from neuron-type-specific ‘synaptic reweighting’ that disrupts E/I balance at both single-neuron and local circuit levels [41, 43]. The imbalance is further shaped by neuroinflammatory signalling driven by activated microglia and astrocytes, which release proinflammatory cytokines and neurotrophic factors [44–49] that modulate synaptic transmission and neuronal excitability. Consequently, chronic pain has been proposed to represent, at least in part, a gliopathy [50]. Notably, heightened neuronal activity itself promotes glial activation [51, 52], establishing a self-reinforcing loop that sustains E/I imbalance and facilitates DH sensitisation, hence pain chronicification. Breaking this pathological cycle requires either (1) reducing neuroinflammation, e.g., nonsteroidal anti-inflammatory drug (NSAID) strategies remain the first-line treatment, acting at both PNS and CNS levels, or (2) restoring E/I balance within DH circuits. The latter has historically been difficult because of the limited ability to manipulate defined neuronal populations with sufficient precision for selective E/I rebalancing. However, rapid advances in single-cell transcriptomics**,** molecular profiling, and the identification of cell-type-specific genetic markers are now enabling targeting of DH neuron classes, providing a foundation for next-generation, mechanism-based therapies aimed at restoring disrupted E/I balance while minimising off-target effects.

## Synaptic Mechanisms of DH Sensitisation

Synaptic plasticity contributes to both the initiation and maintenance of DH sensitisation. It is now well established that chronic pain induces structural and functional reorganisation at both pre- and postsynaptic sites within DH circuits, causing persistent disruption of the balance between excitatory and inhibitory neurotransmission, and, ultimately, sustained network hyperexcitability.

### Morphological Changes

Chronic pain profoundly remodels the DH cytoarchitecture. Studies have demonstrated rapid, activity-dependent dendritic remodelling in the superficial DH (lamina I–II) within 30 min of inflammation onset, accompanied by the formation of spine-like protrusions [53]. These morphological changes are associated with increased amplitude and frequency of spontaneous excitatory postsynaptic currents (sEPSCs), dependent on the activation of AMPA and NMDA glutamate receptors and presynaptic Ca^2+^-channels [53]. The coupling between dendritic remodelling and enhanced excitatory synaptic transmission is further supported by increased dendritic spine density in DH neurons in inflammation, whereas genetic inhibition of spine formation markedly attenuated inflammatory hypersensitivity [54].

Pronounced structural plasticity has also been observed in the deeper DH laminae (IV–V) under neuropathic pain conditions. Following peripheral nerve injury or in painful diabetic neuropathy, lamina IV–V neurons—many of which are WDR neurons—display promoted dendritic spine growth and enlarged spine head diameter [55–58]. These morphological adaptations provide a structural basis for enhanced excitatory synaptic input and sustained network hyperexcitability. Consistently, pharmacological inhibition of dendritic spine formation significantly alleviated neuropathic pain behaviour [55, 56]. Altogether, these support a causal role of morphological plasticity in both superficial and deep DH layers in central sensitisation.

Structural reorganisation in chronic pain occurs not only in excitatory circuits. Converging evidence indicates concurrent degeneration of inhibitory synapses within the DH. A marked reduction in the density of GAD65-immunoreactive (GABAergic) synaptic boutons has been observed in laminae I–II, closely correlating with the time window of developing neuropathic hypersensitivity in the CCI model [59].

Thus, chronic pain induces bidirectional synaptic remodelling within DH circuits, enhancing excitatory connectivity through dendritic spine proliferation, with a concomitant loss or weakening of inhibitory synapses. These complementary structural changes shift the E/I balance toward hyperexcitation driving DH sensitisation.

### Changed Vesicular Neurotransmitter Release

**Fig. 1:**
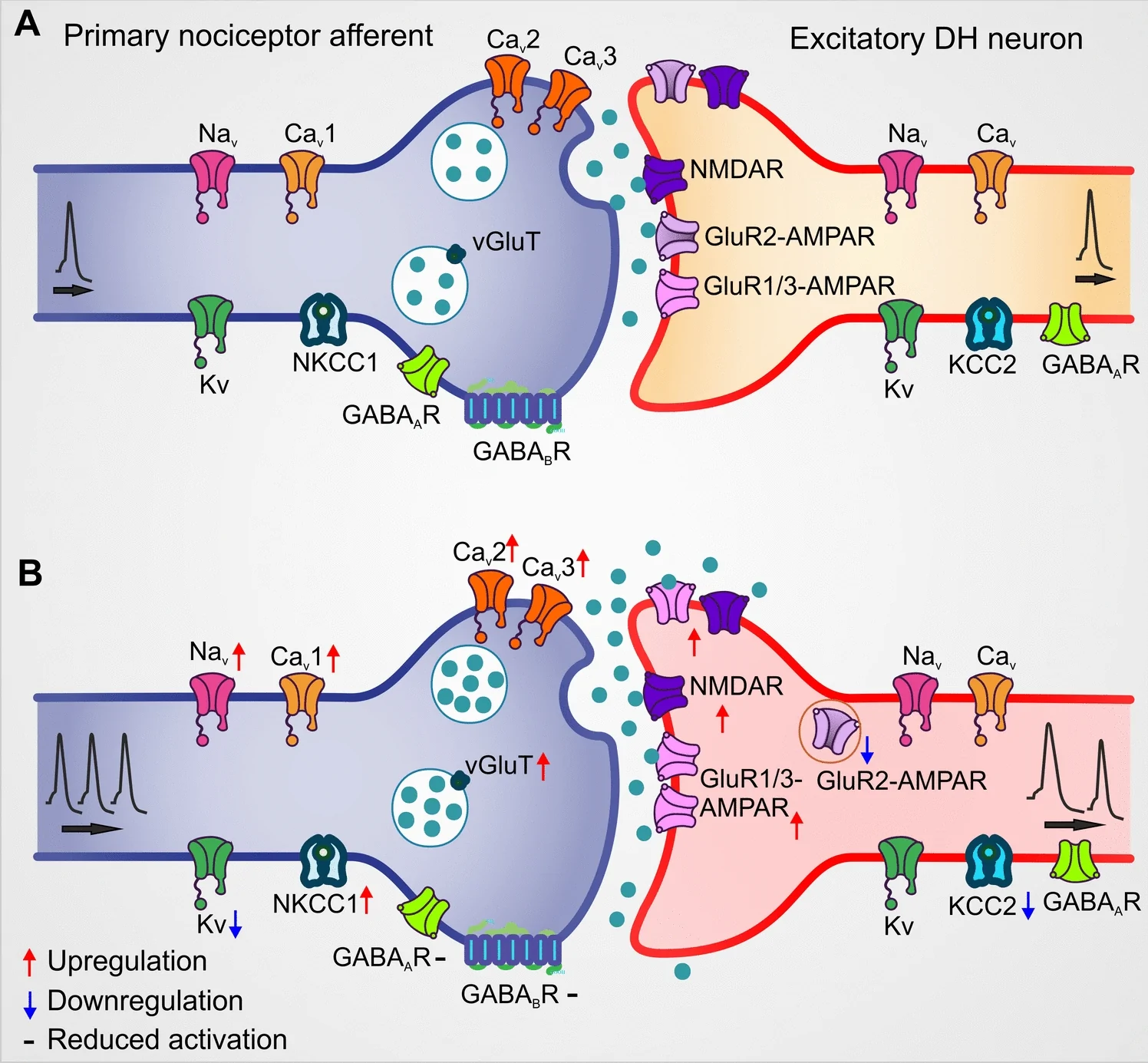
Schematic illustration of a first-order synapse in nociceptive pathways between a primary afferent terminal and an excitatory DH neuron under physiological (**A**) and chronic pain conditions (**B**). Arrows indicate pain-induced changes in the functioning of key molecular and cellular targets that may be the focus of mechanism-based chronic pain therapies aimed at restoring normal synaptic transmission. Schematic elements were created using graphic components obtained from Biodraws.com.

Presynaptic alterations in chronic pain amplify excitatory transmission in the DH by increasing glutamate release, and apparently vesicular glutamate content (Fig. 1). Bee venom injection increased excitatory amino acid levels while decreased inhibitory ones [60]. Similarly, CCI increased synaptosomal aspartate and glutamate levels by  > 40% [61].

The apparent increase in vesicular glutamate content is more readily explained by the altered activity of vesicular glutamate transporter 2 (VGluT2), which determines the quantal size of synaptic vesicles [62]. VGluT2 is expressed in both DH neurons [63] and terminals of nociceptive primary afferents [64], and its expression is upregulated following tissue injury [65]. Importantly, modulating VGluT2 expression attenuates neuropathic and inflammatory hypersensitivity [66], linking enhanced vesicle content directly to central sensitisation.

In addition to increased vesicular loading, chronic pain increases presynaptic release probability by increasing the intrinsic excitability of primary nociceptors. In models of diabetic neuropathy [67–69] and traumatic nerve injury [70–72], nociceptive DRG neurons demonstrated upregulated expression and current density of T-type voltage-gated calcium channels (VGCCs). The channel upregulation lowers the threshold for burst firing [68] and promotes aberrant activity, which in turn facilitates spontaneous and evoked glutamate release from primary afferent terminals in the DH [73]. Consistently, pharmacological blockage or genetic silencing of T-type VGCCs markedly reduced neuropathic pain [74–77], highlighting the channel's role in driving presynaptic hypersensitivity and DH hyperexcitability. The contribution of T-type VGCCs toward excitatory transmission in the superficial DH [69] was also reflected by the effect of selective channel blocker TTA-P2, which reduced the frequency of miniature excitatory postsynaptic currents (EPSCs) in superficial DH neurons but did not measurably alter GABA release in laminae I–II [73]. The effects were strongest in projection neurons, absent in inhibitory interneurons of the superficial DH, and not evident in the deep DH [73], suggesting neuronal-type- and region-specific modulation of glutamatergic afferent inputs.

Collectively, chronic pain enhances presynaptic excitatory drive through two converging mechanisms: (i) increased vesicular glutamate content (consistent with VGluT2 upregulation), and (ii) augmented presynaptic excitability mediated by T-type VGCCs. These synergistic mechanisms increase the amount of glutamate released within the DH, causing sustained excitatory drive and hyperexcitability, thus promoting central sensitisation.

### Glutamate Clearance and Postsynaptic Receptor Changes

Consistent with increased glutamate release, accumulation of extrasynaptic glutamate is a hallmark of central sensitisation. Following SCI, extracellular glutamate can rise dramatically (up to 37-fold) [78], reaching levels capable of inducing excitotoxicity and cell death [79]. Under normal conditions, glial glutamate transporters (GLTs) maintain low extracellular glutamate levels by rapid uptake and recycling. Pharmacological inhibition of GLTs produced thermal and mechanical hypersensitivity and enhanced DH neuronal responses to peripheral stimulation [80], whereas increasing uptake capacity *via* GLT-1 gene transfer alleviated inflammatory hyperalgesia and neuropathic allodynia [81].

Inflammatory and neuropathic pain states impair glutamate clearance through several, partially overlapping mechanisms. The proinflammatory cytokine Il-1β promotes endocytosis of key glial transporters (GLT-1 and GLAST) [82], while chronic constriction [83] and partial sciatic nerve ligation [84] downregulate transporter expression. However, spared nerve injury increases extracellular glutamate without detectable changes in GLT expression [85], suggesting that other regulatory mechanisms are also involved. Importantly, reduced expression of GLT-1 and GLAST also weakens GABAergic transmission [86], coupling impaired glutamate homeostasis to disinhibition of DH neurons, further amplifying DH hyperexcitability.

The combined effect of increased glutamate release and reduced glial uptake results in glutamate spillover, which broadens signalling beyond the synaptic cleft and promotes activation of peri- and extrasynaptic glutamate receptors, driving neuronal hyperexcitation (Fig. 1). Both NMDA and AMPA receptor (AMPAR) subtypes contribute to this amplification [84, 87–90]. Persistent pain promotes insertion of Ca^2^⁺-permeable, GluR1-containing AMPARs at synapses between primary nociceptors and DH neurons, causing sustained depolarisation, enhanced Ca^2^⁺-influx, and strengthening excitatory drive [91] that correlates closely with the development and maintenance of inflammatory hyperalgesia [91]. As extracellular glutamate rises and spillover becomes more prominent, these changes also increase recruitment of extrasynaptic AMPARs [92, 93], extending spatially and temporally excitation of DH neurons into a hyperresponsive state.

Thus, chronic pain disrupts glutamatergic homeostasis at multiple levels: by increasing vesicular glutamate content and release probability, impairing clearance mechanisms (glial uptake), promoting activation of extrasynaptic receptors, and shifting receptor composition to favour sustained neuronal excitation, hence the persistence of central sensitisation.

## Imbalanced Synaptic E/I in DH Sensitisation

### Pain-Induced Synaptic Excitation of DH Neuron Types

Potentiation of glutamatergic transmission within DH circuits is a consistent and well-established hallmark of chronic pain. This long-term potentiation arises from enhanced primary afferent drive and augmented synaptic excitation of spinal neurons. WDR neurons become sensitised after SCI [94] and display increased spontaneous firing in inflammatory and neuropathic pain models [95, 96]. Spontaneous activity of A- and C-fibres, a form of aberrant peripheral input in chemotherapy-induced pain [97], chronic inflammation [98], diabetic neuropathy [99, 100], and peripheral nerve injury [101–103] can both initiate and maintain chronic pain. At the circuit level, facilitated synaptic transfer from C-fibre afferents to projection neurons can induce central sensitisation [104], long-term potentiation (LTP) at DH synapses [105], a plausible mechanism by which low-frequency nociceptor activity is converted into persistent amplification of nociceptive signalling. This also engages non-neuronal cell types, such as activation of spinal microglia [106].

Synaptic potentiation of DH excitability in persistent pain is highly neuron-type-specific. This neuron-type specificity is particularly clear in lamina II, where Complete Freund’s adjuvant (CFA)-induced peripheral inflammation differentially changed spontaneous and miniature EPSCs (sEPSCs and mEPSCs) in excitatory versus inhibitory interneurons. Adapting-firing lamina II neurons show increased sEPSC and mEPSC frequency, indicating enhanced glutamate release (both action-potential-dependent and -independent) from primary afferents and local DH interneurons, whereas tonic-firing (predominantly inhibitory) interneurons displayed reduced sEPSC frequency under the same conditions [41]. A similar bidirectional pattern was reproduced by the proinflammatory cytokine interleukin 1β (IL-1β), which increased excitatory synaptic drive (i.e., sEPSC frequency) onto excitatory, delay-firing DH neurons, but reduced it onto inhibitory, tonic-firing neurons [107].

Comparable cell-type-specific changes occur in neuropathic pain. After peripheral nerve injury, excitatory neurons (delay, transient, and irregular firing types) showed increased sEPSC and mEPSC frequency, whereas inhibitory (tonic-firing) neurons exhibited reductions [22, 23]. Interestingly, brain-derived neurotrophic factor (BDNF), released by activated microglia following nerve injury [50, 108], can mimic such patterns of altered glutamatergic transmission [109], further implicating neuron-glia signalling in shaping E/I imbalance.

Postsynaptic strengthening of excitatory drive further consolidates DH sensitisation and aligns with a form of LTP at DH synapses. There is less consistency between pain models, likely because pain severity influences the magnitude and profile of postsynaptic changes. In CFA-induced inflammation [41] and IL-1β exposure [107], sEPSC amplitude increased robustly in excitatory but not in inhibitory neurons. In neuropathic pain models, sEPSC amplitude increased in excitatory neurons while reduced in inhibitory neurons [22], whereas sciatic nerve axotomy produced weaker or absent postsynaptic effects [23], potentially reflecting reduced inflammation and diminished microglial BDNF release, which was found to decrease sEPSC amplitudes in inhibitory neurons [109].

**Fig. 2:**
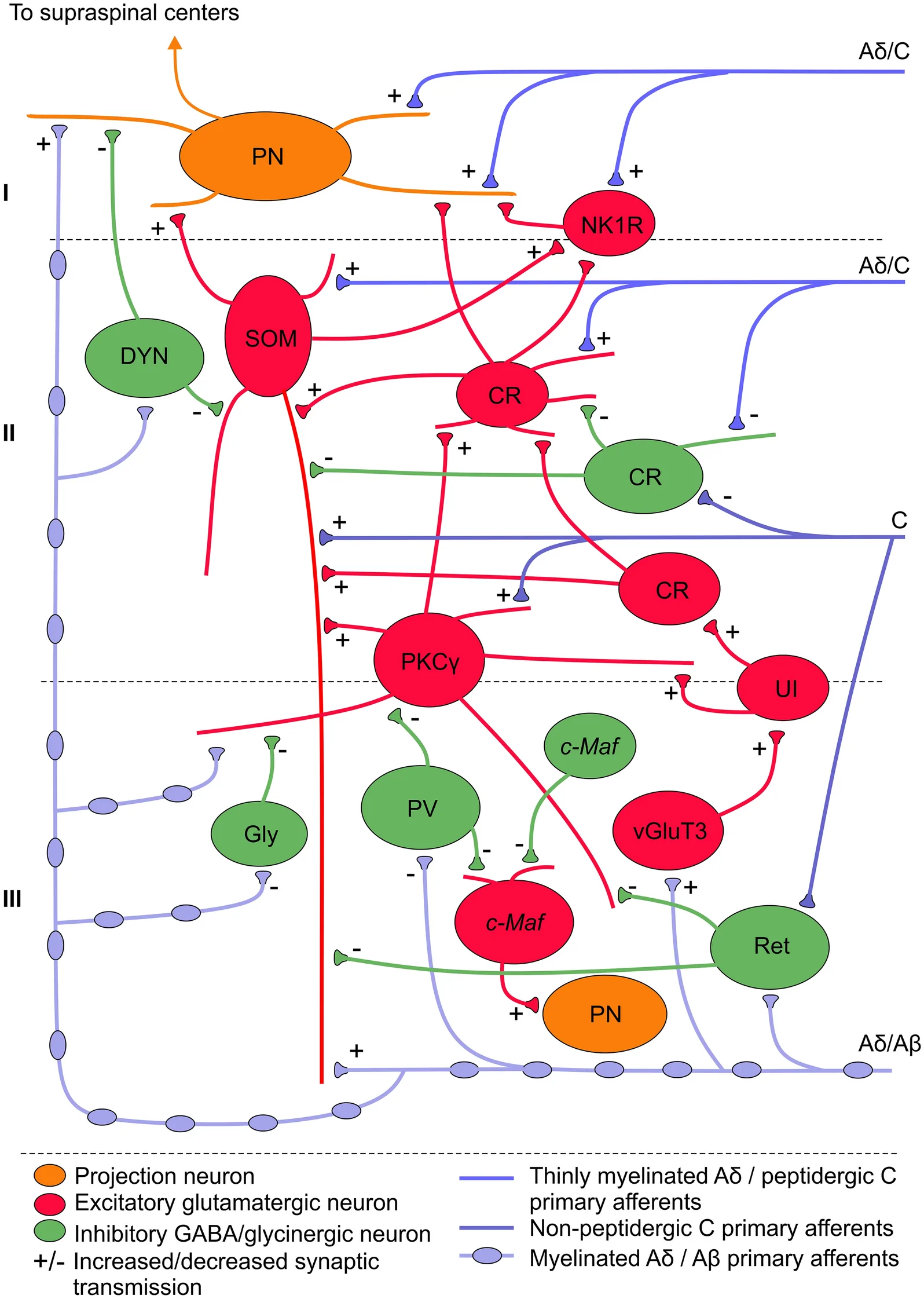
Chronic pain-induced E/I imbalance in the DH sensitisation. Simplified illustration depicts the organisation of DH circuitry, indicating the changes in excitatory and inhibitory synaptic transmission in chronic pain conditions. Abbreviations: NK1R, neurokinin 1 receptor; SOM, somatostatin; CR, calretinin; DYN, dynorphin; VGLUT3, vesicular glutamate transporter 3; PKCγ, protein kinase C gamma; PV, parvalbumin; Ret, retinin; Gly, glycinergic neurons; UI, unidentified. The scheme was adapted from [268–270].

These experimental findings demonstrate bidirectional, neuron-type-specific remodelling of excitatory synaptic transmission, both pre- and postsynaptic alterations, within the DH. Ultimately, excitatory DH neurons receive potentiated glutamatergic input, while inhibitory DH neurons experience suppressed excitatory drive. This asymmetric modulation diminishes inhibitory recruitment and shifts the E/I balance toward hyperexcitation (Fig. 2), eventually causing the DH sensitisation and chronic pain.

### Reduced Synaptic Inhibition—GABAergic Changes

A second pillar of E/I imbalance within the DH circuits is reduced GABAergic synaptic inhibition, which contributes to the development and maintenance of allodynia, hyperalgesia, and spontaneous pain [110–114].

Early hypotheses emphasised that the loss of DH inhibition results from cell death of inhibitory neurons through degeneration [110, 115]. Experimental ablation of inhibitory neurons could indeed elicit pain-like behaviour [116], and SCI has been associated with the loss of GABAergic interneurons [117] and reduced GABAergic markers (GAD65 and GAD67) [118]. In neuropathic pain models, however, selective loss of GABAergic or glycinergic neurons is not consistently observed [112, 119, 120], and neither gross loss of GABA/GABA_A_ receptors [110, 120, 121] nor a reduction in excitatory synapse number onto GABAergic neurons [122] is a requirement for pain hypersensitivity. This convergence argues that functional reconfiguration in synaptic inputs to inhibitory neurons—rather than widespread neuronal loss—underlies disinhibition and the DH hyperexcitability.

One prominent mechanism is a weakened excitatory drive onto GABAergic interneurons. In neuropathic conditions, GABAergic lamina II neurons receive reduced primary afferent inputs, as evidenced by a decreased mEPSC frequency and an increased paired-pulse ratio of Aδ- and C-fibre-evoked responses [122], suggesting decreased presynaptic release probability. Although the retrograde messenger mediating this presynaptic depression remains unknown, its dependence on intracellular calcium supports an activity-driven form of synaptic suppression that selectively targets inhibitory ‘gate’ neurons. The reduction in primary afferent input may also reflect loss of synapses onto GABAergic neurons, in line with the observed decrease in GAD65-positive axonal boutons within laminae I–II after CCI, which correlated closely with the time course of pain behaviour [59].

The reduction in excitatory recruitment of GABAergic neurons contrasts with the strengthened glutamatergic connectivity onto excitatory neurons, sharpening the net E/I shift. Cell-type-specific divergence may further magnify this imbalance. Dorsal root stimulation paradigms (low- or high-frequency stimulation) can induce long-term depression (LTD) in GABAergic neurons while producing LTP in spinothalamic projection neurons, even though both forms depend on NMDAR-driven intracellular Ca^2+^ rises [52]. These highlight that identical signals can be translated into opposite synaptic outcomes depending on postsynaptic cell identity and explain how chronic pain produces coordinated cell-type-specific circuit reconfiguration rather than uniform synaptic potentiation within the DH.

Disinhibition of DH interneurons is also reinforced presynaptically through activity-dependent suppression of inhibitory transmitter release, representing the other mechanism characteristic of various pain states. Increased C-fibre activity elevates glutamate release that recruits group I metabotropic glutamate receptors (mGluRs) [123] and neurokinin-1 (NK1) receptors sensitive to substance P [124]; both receptors can promote endocannabinoid synthesis, which retrogradely suppresses GABA and glycine release from presynaptic terminals [123, 125, 126]. This mechanism links heightened nociceptor input directly to reduced inhibitory tone in the DH, creating a feedforward loop in which increased excitation weakens its own inhibitory constraints.

Further to presynaptic inhibition, reduced GABAergic inhibition can additionally facilitate excitatory transmission by upregulating NMDAR function. Mechanistically, loss of inhibition can promote PKA-dependent phosphorylation and synaptic accumulation of NMDARs [127], shifting the NMDAR current-voltage relationship toward more negative potentials [128], thereby facilitating A-fibre-mediated excitation in superficial DH microcircuits [129]. Such facilitation is particularly relevant for lamina I projection neurons, as reduced inhibition can unmask or strengthen low-threshold Aβ-fiber input [130], providing a circuit-level route of aberrant pain perception to develop tactile allodynia.

Summarising, pain-induced disinhibition originates from functional changes in GABAergic transmission at presynaptic, postsynaptic, and circuit levels. The weaken excitatory drive onto inhibitory neurons can retrogradely suppress GABA release and reorganise synaptic connectivity within DH circuits, ultimately reducing inhibitory control and imbalancing the E/I toward hyperexcitation and DH sensitisation (Fig. 2).

### Reduced Glycinergic Tone in DH Circuits

Alongside GABAergic, diminished glycinergic inhibition contributes to the DH disinhibition and central sensitisation in inflammatory and neuropathic pain. This mechanism is especially prominent in superficial nociceptive circuits and involves both presynaptic (glycine release) and postsynaptic changes (glycine receptor (GlyR) function), with a clear neuronal-type specificity.

Glycine is considered the primary inhibitory neurotransmitter in lamina I [131, 132], and pain states significantly reduce the frequency of GlyR-mediated miniature inhibitory postsynaptic currents (mIPSCs) in lamina I neurons [126]. Inflammatory pain sensitisation is tightly linked to pro-nociceptive mediator prostaglandin E2 [133], which activates PKA and drives phosphorylation of α3 subunit-containing GlyRs [126, 134, 135], enriched predominantly in the superficial DH inhibitory synapses [135]. The resulting reduction in glycinergic tone, therefore, contributes directly to circuit disinhibition and pain hypersensitivity.

Glycinergic dysfunction also extends to deeper DH laminae and contributes to pathological cross-laminar coupling. After nerve injury, suppression of feedforward glycinergic inhibition onto PKCγ-positive neurons can facilitate the recruitment of nociceptive pathways by low-threshold, non-nociceptive A-fibre inputs [136]. Spinal nerve ligation reduced Aβ-fiber-mediated EPSCs in lamina III glycinergic neurons, diminishing their inhibitory output to PKCγ-positive cells [136]. In turn, EPSPs from PKCγ-positive neurons to nociceptive lamina II transient central cells are potentiated, boosting activation of pain pathways by innocuous Aβ-fiber input (Fig. 2). This maladaptive excitation aligns with the NMDAR-independent spread of excitation from deep to superficial DH in neuropathic animals [137]. Such cross-laminar excitation, absent in physiological conditions, is thought to represent the mechanistic basis underlying mechanical allodynia, where normally non-painful stimuli become painful. The loss of glycinergic tone may be a mechanism of the heightened WDR neuron activity in the deep DH after SCI [138, 139], further amplifying DH hyperexcitability.

The reduction in glycinergic inhibition appears to be cell-type specific. While excitatory DH neurons receive reduced glycinergic input, inhibitory neurons display strengthened glycinergic transmission. IL-1β increased glycinergic IPSCs in lamina II GABAergic neurons, producing a form of glycinergic LTP [139]. Given that glycine also acts as a co-agonist of NMDARs, known as pro-nociceptive receptors in the superficial DH, such potentiation may further activate NMDARs through glycine spillover [140] that, in turn, amplifies excitatory drive within DH circuits. Consistent with this, increased c-Fos expression in glycinergic neurons correlates with glycine-mediated LTP in pain states [141], indicating neuronal hyperactivity rather than silencing.

Together, these suggest that glycinergic neurons do not lose their function but instead redistribute their inhibitory outputs—reducing transmission to excitatory neurons while enhancing it to inhibitory interneurons—further biasing the network E/I imbalance toward hyperexcitation.

### Dysregulated Chloride Gradient and KCC2 Downregulation

In addition to the mechanisms described above, DH disinhibition is further amplified by dysregulation of the transmembrane chloride gradient, which determines whether GABA_A_R and/or GlyR activation allows chloride influx. It results in hyperpolarising cells (inhibition) or, paradoxically, depolarising cells (disinhibition). Effective inhibition requires the maintenance of low intracellular chloride concentration ([Cl⁻]ᵢ), controlled largely by the potassium-chloride cotransporter 2 (KCC2), which extrudes Cl⁻ ions from neurons to maintain a low [Cl⁻]ᵢ [142, 143]. Under physiological conditions, the chloride reversal potential is below the resting membrane potential, ensuring that GABAergic and glycinergic transmission is inhibitory. In multiple pain models, including inflammation [144], nerve injury [113], SCI, and painful diabetic neuropathy [145, 146], KCC2 expression and/or function is downregulated in the DH (Fig. 1) [147–149], elevating [Cl⁻]ᵢ and shifting the chloride reversal potential toward more depolarised values. This shift reduces inhibition or, when sufficiently severe, can convert inhibitory responses to GABA and glycine into depolarising (excitatory) events [100, 126, 150] that disrupt inhibitory control even if GABA and glycine release remains unchanged. KCC2 downregulation often coincides with increased GABA levels (e.g., in diabetic neuropathy) [147, 148], resulting in GABA_A_R-mediated currents becoming less hyperpolarising, which causes disinhibition and hyperexcitability in the DH [151].

Activation of microglia is heavily implicated in KCC2 downregulation in pain conditions through microglial release of BDNF, acting on tropomyosin-related kinase B (TrkB) receptors expressed by DH neurons [152]. The BDNF-TrkB pathway triggers two major intracellular signalling cascades [153, 154], involving the Shc and the PLCγ pathways in neuronal KCC2 downregulation.

Chloride dysregulation has consequences beyond ‘weaker inhibition’. Elevated [Cl⁻]ᵢ can cause paradoxical excitation, where depolarising GABA_A_R or GluR responses can facilitate NMDAR activation by relieving Mg^2^⁺ block, coupling disinhibition directly to excitation. This has been demonstrated in persistent inflammation, with GABAR agonists paradoxically exacerbating pain hypersensitivity, while antagonists produced antinociceptive effects [155]. Restoring KCC2 function (or targeting upstream pathways) holds therapeutic potential to normalise inhibitory efficacy of GABAergic and glycinergic transmission, potentially normalising network gain without requiring large-scale suppression of excitatory transmission.

## Neuron-Type-Specific Strategies for Developing Mechanism-Based, Side-Effect-Free Therapies

Both excitatory and inhibitory DH neurons exhibit marked heterogeneity in morphology, their intrinsic excitability, and afferent innervation. Over the past 15 years, considerable effort has focused on defining discrete DH neuron identities along multiple axes, including somatodendritic architecture, firing patterns, laminar position, neurochemical phenotype, and gene-expression signature, aimed to clarify how specific populations contribute to nociceptive processing. Single-cell RNA sequencing in the mouse DH has identified 15 excitatory and 15 inhibitory neuronal subtypes [156], indicating a range of cellular diversity comparable to that of cortical circuits. Consistent DH taxonomies have now been described in mouse [157], macaque [158], and human spinal cord [159], supporting the cross-species conservation of this organisation.

### Nociceptive DH Neuron Classes Altered in Pain States

By integrating genetic clustering with activity-dependent gene expression profiling, it has been shown that distinct DH neuron populations make differential contributions to nociceptive processing [160], with galanin-positive interneurons emerging as a major inhibitory ‘gate’ population. Molecular markers have now been established for several other key nociceptive-processing neuron populations. Selective ablation experiments identified a pathway for processing mechanical pain that comprises excitatory somatostatin-positive (SOM) and inhibitory dynorphin-positive (Dyn) neurons [161]. Dyn-lineage interneurons gate A-fibre input onto SOM cells, which provide drive to NK1R-expressing neurons in laminae I–II (Fig. 2), including projection neurons that transmit nociceptive signals to supraspinal centres [18]. In chronic pain conditions, disinhibition of SOM neurons facilitates polysynaptic A-fibre-mediated input to downstream circuits; conversely, conditional knockdown of T-type VGCCs in SOM neurons attenuates both thermal and mechanical hypersensitivity [162]. SOM neurons are also embedded within a circuitry formed by calretinin-expressing (CR) interneurons. Inhibitory CR neurons gate nociceptive primary afferent input to SOM cells [163], whereas excitatory CR neurons constitute an intrinsic excitatory network that enhances glutamatergic drive to both SOM- and NK1R-positive neurons and lamina I projection neurons [164].

Upstream of the SOM population, PKCγ-expressing excitatory interneurons in inner lamina II/lamina III play a key role in amplifying Aβ-fibre inputs. Under physiological conditions, PKCγ neurons are largely kept silent by inhibitory gating from three lamina III interneuron populations: parvalbumin-positive (PV) [165–168], early tyrosine kinase-positive (RET) [169], and glycinergic neurons [136, 167]. Neuropathic pain disrupts this inhibitory control [136, 167], allowing Aβ-fibre inputs to recruit PKCγ neurons. Concurrent activation of VGluT3-expressing neurons further increases excitatory tone in the DH and drives excessive activation of both PKCγ- and CR-expressing interneurons [170, 171]. In addition, activation of c-Maf-expressing excitatory neurons, synaptically connected to lamina III–IV projection neurons, has been implicated in neuropathic pain [172].

The recruitment of specific DH neuron populations is strongly influenced by injury aetiology, and neuron molecular identity predicts the pain signal modalities they convey. For example, lamina II_i_ CR neurons preferentially mediate inflammation-induced mechanical allodynia, whereas lamina II/III PKCγ interneurons are primarily engaged in neuropathic mechanical allodynia [173]. Laminae III–IV VGLUT3 neurons contribute to both inflammatory and neuropathic pain, mediating dynamic but not punctate allodynia [171], while cholecystokinin-positive (CCK^+^/VGLUT3^-^) neurons participate in both modalities [173]. Inhibitory DH neuron populations also play distinct roles: RET-expressing neurons in laminae III–V are critical for both inflammatory and neuropathic pain and regulate thermal as well as mechanical sensitivity [169], whereas PV-positive interneurons selectively gate mechanical input and primarily shape neuropathic mechanical hypersensitivity [165].

Despite recent progress in mapping nociceptive-processing DH populations (Table 1), detailed information on how each subtype is altered in chronic pain remains limited. Nonetheless, the emerging molecular and functional taxonomy of DH neurons provides a powerful framework for developing cell-type-specific interventions that would selectively rebalance E/I within pain-relevant microcircuits. Such strategies could involve a) strengthening or restoring recruitment of inhibitory ‘gate’ populations, b) dampening sensitised excitatory interneurons, and c) normalising projection-neuron gain, with the overarching goal of correcting circuit dysfunction while minimising off-target effects.

**Table 1:** Summary of chronic-pain-induced changes observed in defined DH neuron subpopulations.

**Functional role**	**Neurotransmitter**	**Molecular/genetic identity**	**Lamina**	**Pain model**	**Pain-induced changes**	**Methodology**	**Ref**
Projection neurons	Glutamate		I	SNI	Increased AP output in response to low-threshold stimuli	Patch clamp	[239]
				Capsaicin-induced allodynia		2-photon calcium imaging	[259]
				SNI	Increased AP output. Postsynaptic disinhibition due to microglia-mediated degradation of perineural nets	Patch clamp	[260]
Excitatory interneurons	Glutamate	PKCγ	II–III	SNL	Decreased feed-forward synaptic inhibition by lamina III glycinergic neurons. Increased excitatory synaptic transmission to lamina II neurons in response to Aβ-fibre stimulation	Patch clamp	[136]
				SCI	Increased activation	c-Fos IHC	[261]
		Tac1*	I–II	SNI	Increased activity	scRNA seq	[160]
		Tacr1*					
		CGRP	III	SNI	Unsilencing in response to an innocuous mechanical brush	c-Fos IHC, patch clamp, chemogenetics	[262]
		*VGlut3*	II–III	SNI	Increased Aβ-fibre-driven synaptic input. Augmented Aβ-fibre-driven AP output to lamina I-II neurons	Patch clamp, targeted ablation	[171]
		CR	II–III	SCI	Increased activation in response to mechanical stimulation	c-Fos IHC	[261]
				SNI	Increased activation	c-Fos IHC	[165]
		CCK	II–III	CCI	Increased activation. Enhanced AMPAR-mediated postsynaptic currents.	c-Fos IHC, patch clamp	[263]
		NPY1R	I–II	SNI	Enhanced membrane excitability (augmented firing frequency). Increased amplitude and frequency of postsynaptic excitatory currents.	Patch clamp	[264]
		NK1R*	I–II		Increase AP output. Decreased postsynaptic inhibition (*circFhit*-mediated reduced GAD65 expression)	Patch clamp, optogenetics	[265]
Inhibitory interneurons	Glycine		III	SNL	Decreased synaptic transmission from Aβ primary afferents.	Patch clamp	[136]
	GABA/glycine GABA/glycine	PAX2	II–III	SCI	Decreased activation	c-Fos IHC	[261]
		PV	III	CCI	Decreased glycine release	ErbB4 siRNA	[266]
					Alteration of firing pattern: switch from high-frequency tonic firing to adaptive firing	Patch clamp	[166]
				SNI	Retinoic acid receptor (RARα)-mediated reduction of pre- and postsynaptic inhibition of lamina II-III excitatory (PKCγ^+^ and vGluT3^+^) interneurons by PV^+^ neurons. RARα-mediated reduction of postsynaptic inhibition of excitatory lamina II PV^+^ neurons.	Patch clamp, optogenetics	[267]
					Loss of inhibitory synapses in PKCγ^+^ neurons	IHC	[165]
	GABA	*Gal*	I–II	SNI	Reduced recruitment to noxious thermal and mechanical stimuli	scRNA seq, behavioural, chemogenetics	[160]

Pain models: SCI, spinal cord injury; SNL, spinal nerve ligation; SNI, spared nerve injury; CCI, chronic constriction injury. All studies were conducted in mice, except for refs 136 and 239, which used rats. * NKR1, *Tacr1,* and *Tac1* are also expressed in projection neurons.

Abbreviations: CCK, cholecystokinin; CGRP, calcitonin gene-related peptide; CR, calretinin; Gal, galanin; IHC, immunohistochemistry; NK1R, neurokinin receptor type 1; NPY1R, neuropeptide Y receptor type 1; PKCγ, protein kinase C gamma; PV, parvalbumin; vGLuT3, vesicular glutamate transporter type 3.

### AMPAR-Targeting Approaches

Fast excitatory synaptic transmission in the DH is mediated primarily by AMPARs, positioning these receptors as key effectors of both the induction and persistence of chronic pain [91]. Consistent with this role, AMPARs have been extensively explored as potential therapeutic targets, with the proven remarkable antinociceptive [174–179] and antipruritic [180] effects of AMPAR antagonists in preclinical models. Their clinical translation has been, however, constrained by dose-limiting adverse effects, including motor impairment, dizziness, ataxia, and others. A central limitation of systemic AMPAR blockade rests in its lack of cell-type and circuit specificity: inhibiting AMPARs on excitatory neurons is expected to be analgesic, whereas suppressing AMPARs in inhibitory interneurons can weaken inhibition, thereby enhancing pain signalling [126, 181]. This dichotomy motivates a shift from global AMPAR antagonism toward strategies that modulate AMPAR function selectively within defined DH neuron populations, reducing excessive excitatory drive while preserving or even strengthening inhibitory control.

Genetic and molecular modifications provide a versatile approach to this precision by targeting (1) AMPAR subunits directly, (2) auxiliary proteins that modify receptor trafficking and synaptic stabilisation, and (3) associated kinases and scaffolding proteins that govern AMPAR motility and function, tuning the receptor properties in specific neuronal populations.

Pathological plasticity across multiple pain models is accompanied by an increased contribution of Ca^2^⁺-permeable AMPARs at nociceptive synapses, conceptualised as a shift toward surface expression of the GluA1-containing AMPAR assemblies that enhance Ca^2^⁺ entry and facilitate LTP-like strengthening of excitatory transmission [88, 174, 182, 183] while reduced GluA2-containing AMPAR [88] (Fig. 1). Mechanistically, several molecular mechanisms contribute to the AMPAR trafficking shift. First, upregulation of the auxiliary protein α2δ1 [184] disrupts normal GluA1/GluA2 assembly, favouring Ca^2+^-permeable AMPAR accumulation [185]. Second, phosphorylation-dependent removal of GluA2 from the synaptic membrane provides a direct route to Ca^2+^-permeable AMPAR enrichment: protein kinase C (PKC)-driven phosphorylation of GluA2 at Ser880 is widely implicated in GluA2 trafficking and internalisation that remodels AMPAR composition both at the synapses and extrasynaptic membranes. Pharmacological inhibition or PKCα knockdown alleviates inflammatory pain [93, 186]. Third, kinases that act primarily on GluA1, i.e., PKCγ [187, 188], PKA [182], and CaMKII [189], can promote membrane insertion and stabilisation of AMPARs. Fourth, scaffolding proteins, such as PICK1 and AKAP150, facilitate kinase-receptor interactions that control AMPAR phosphorylation state and localisation; disrupting these complexes pharmacologically or by genetic deletion has been shown to reduce pathological AMPAR regulation and alleviate chronic pain in models of inflammatory and neuropathic pain [190–192]. Finally, transmembrane AMPAR regulatory proteins (TARPs) modify receptor trafficking and promote synaptic translocation. For example, TARP-γ2 is required for recruitment of Ca^2+^-permeable AMPARs at primary afferent-DH synapses [193], and antagonism of TARP-γ8 yields antinociceptive effects [194].

These findings identify multiple molecular points for AMPAR-targeted interventions applied with cell-type specificity to restore E/I balance rather than blocking AMPAR currents indiscriminately. Interventions can be designed to target:

1. α2δ1 auxiliary protein;
2. Kinases phosphorylating the receptor subunits;
3. Scaffolding proteins coupling kinases to AMPARs;
4. TARP family;
5. AMPAR subunit composition (e.g., tuning the GluA1/GluA2 ratio or deploying engineered mutant subunits).

#### α2δ1 Protein, Kinases and Kinase-interacting Proteins

Downregulation of α2δ1, kinases, and kinase-interacting proteins can be applied either globally (in all neuronal types) or restricted to excitatory DH neurons using cell-specific promoters. Reducing α2δ1 protein expression restores normal assembly of Ca^2+^-impermeable AMPARs and disrupts pathological α2δ1-NMDAR complexes in DH neurons and central terminals of primary afferents, alleviating pain [195, 196].

A key translational consideration is that many AMPAR-regulatory kinases (e.g., PKC isoforms, the major kinases involved in regulating AMPAR assembly) affect the receptor functioning in both excitatory and inhibitory neurons [197, 198]. Cell-type-selective gene delivery, therefore, is central to targeting PKC or its interacting proteins, PICK1 and AKAP150, to prevent undesired network effects. Restricting knockdown to excitatory DH populations using promoters such as vGluT2 could dampen pathological excitation. On the contrary, targeted enhancement of AMPAR-mediated drive in inhibitory populations (e.g., using GAD65- or vGlyT2-driven expression) can restore inhibitory recruitment where it is selectively weakened.

#### TARPs

Manipulating TARPs provides another strategy for selective AMPAR modulation, as TARPs shape both AMPAR trafficking and channel-gating properties in an isoform-dependent manner. TARPs regulate synaptic targeting and stabilisation of AMPARs and can enhance receptor function, including effects on conductance and other biophysical parameters, thereby tuning synaptic strength [199, 200].

Among the best-characterised isoforms, TARP γ2 and γ8 can control AMPAR density at synapses, increase single-channel conductance, and promote receptor insertion into the plasma membrane. Consistent with the concept that TARP identity and stoichiometry help determine AMPAR conductance at a given synapse, γ8-containing complexes can shift AMPAR gating toward higher-conductance states, providing a mechanistic basis for how TARPs can increase synaptic gain beyond simple receptor number [201, 202]. The key challenge remains that TARPs are not restricted to a single class of DH neurons, and manipulating TARPs requires a cell-type-specific approach that would aim to downregulate pro-excitatory TARPs in excitatory DH neurons (using GluT2 promoter) to limit pathological excitation, while upregulating TARP-dependent AMPAR efficacy in inhibitory neurons (*via* GAD65 or vGlyT2 promoters) to strengthen inhibitory transmission and stabilise E/I balance. More refined targeting would be possible with the further mapping of specific TARP expression patterns across defined DH neuron populations, considering that transcriptomic atlases indicate the heterogeneity of auxiliary-subunit assemblies across interneuron classes.

#### Cell-specific Targeting of AMPAR Subunits

Manipulation of AMPAR subunits can follow a similar strategy described for TARP targeting: downregulation in excitatory DH neurons and upregulation in inhibitory neurons. Implementing this, however, requires precise targeting, given the heterogeneity of AMPAR subunit distribution across the DH laminae and neuronal types, and because pain states induce remodelling of both subunit composition and membrane trafficking [198].

Among excitatory neurons, projection neurons and PKCγ-positive glutamatergic interneurons, strongly implicated in allodynia [203], are particularly attractive candidates. Downregulation of GluA3 expression, which dominates spinal GluA2/3-containing AMPAR composition [204], is promising because GluA3 is preferentially detected in deeper DH laminae (III–V), which are major termination zones for low-threshold afferents and tactile-to-pain ‘re-routing’. The enrichment of GluA3 in deeper DH supports that GluA3 downregulation in selected excitatory populations may attenuate A-fibre-induced activation of PKCγ-positive neurons in neuropathy [136], thereby relieving allodynia.

NK1R-positive neurons represent another target, as they integrate substance P-dependent nociceptive signalling and include projection neurons critical for ascending pain transmission. In the DH, GluA1 expression overlaps with NK1R [204], and selective downregulation of GluA1 could suppress excitatory transmission and decrease overall DH hyperexcitability. Similarly, targeting GluA3 or GluA4 subunits in NK1R-positive projection neurons of lamina III and IV [205] may further dampen nociceptive drive and provide pain ablation.

Lamina I projection neurons are functionally heterogeneous [18, 206, 207], providing advantageous selectivity for more precise interventions. Two populations of NK1R-expressing lamina I projection neurons with distinct AMPAR subunit profiles (large GluA4⁺/GluA1⁻ and small GluA1⁺/GluA4⁻ neurons) have been described [208], suggesting that subunit-specific targeting could be tailored to projection-neuron subpopulations rather than applied uniformly. Recent work [156] has highlighted Lypd1 as a marker highly expressed in many lamina I spinoparabrachial projection neurons, although Lypd1 expression is not strictly confined to projection neurons within the superficial DH. In a therapeutic context, Lypd1 (potentially in combination with other markers) could support more selective delivery of constructs designed to reduce GluA1- or GluA4-dependent excitation in defined lamina I neurons, providing highly selective analgesia across multiple pain origins. As lamina I projection neurons also transmit itch signals [206], this targeting could be valuable for treating chronic itch.

In inhibitory neurons (GAD65- or vGlyT2-positive populations), the goal is conceptually opposite—enhancing their excitatory recruitment by increasing GluA2 surface expression. As pain upregulates Ca^2+^-permeable-AMPARs in inhibitory neurons [197], restoring GluA2 surface expression would shift AMPAR assemblies toward Ca^2+^-impermeable receptors. Combining subunit engineering with auxiliary-subunit control *via* co-expression of GluA2 with TARP-γ2 or TARP-γ8 to further facilitate membrane trafficking and gating could be used to promote appropriate receptors in inhibitory interneurons. A tandem construct combining GluA2 with a TARP variant may ensure proper receptor assembly while preventing TARP binding to Ca^2+^-impermeable AMPARs.

#### Engineering AMPAR Mutants

Recent studies have identified multiple missense mutations in *GRIA1-4* genes, causing AMPAR channelopathies that are linked to neurodevelopmental or epileptic disorders [209–213]. The mutant variants of GluA1-4 subunits demonstrate changes in AMPAR biophysical properties, including single-channel conductance and open probability, steady-state current, and deactivation kinetics, for homomeric or heteromeric AMPAR. Functionally, all missense *GRIA* variants can be grouped into gain-of-function (GoF), loss-of-function (LoF), or neutral. Selective expression of mutant AMPAR subunits – either GoF or LoF variants can be repurposed for therapeutic gene delivery in chronic pain therapy.

Expression of LoF variants in excitatory DH neurons (e.g., projection or PKCγ-positive neuron populations) could reduce their aberrant excitatory output and eventually lower the DH hyperexcitability. Conversely, GoF variants expressed in inhibitory neurons, such as galanin- or parvalbumin-positive populations, could enhance their synaptic activation and inhibitory tone. This concept of ‘designer channelopathies’ could be customised to specific DH neuron subtypes implicated in a given pain condition, such as targeting PKCγ-positive neurons that predominantly express GluA2/GluA3 heteromers, or projection neurons, expressing mainly GluA1 or GluA4, *via* gene transfer of missense *GRIA1-4*.

Because the effects of GoF and LoF mutations vary in strength, their selection could be adjusted according to the chronic pain state and the degree of E/I imbalance. Controlled, neuron-type-specific expression of mutant AMPAR subunits thus offers a promising strategy to counteract maladaptive AMPAR-driven excitability and restore the E/I balance within the DH circuits in chronic pain states.

### Targeting Inhibitory Tone

Numerous missense variants have recently been identified in genes encoding subunits of GABARs [214–220] and GlyRs [221–226], opening new possibilities to restore impaired inhibitory transmission within DH circuits. Conceptually, the designed genetic interventions could be similar to those proposed for mutant AMPAR variants. Expression of GoF GABAR or GlyR subunits in excitatory DH neurons could compensate for diminished inhibitory input, whilst LoF variants expressed in inhibitory neurons might protect against excessive suppression by descending supraspinal GABAergic tone [227].

A major challenge in restoring inhibitory tone involves the chloride gradient dysregulation, which causes GABAergic or glycinergic signalling to switch from hyperpolarising to depolarising [155]. Restoring KCC2 expression in DH neurons helps re-establish chloride homeostasis, strengthen inhibitory tone, and reduce pain in experimental models [228, 229].

Optogenetic or pharmacological activation of KCC2 has been shown to restore hyperpolarising inhibition, normalise neuronal excitability, and reverse pain hypersensitivity, underscoring the critical role of KCC2 in maintaining spinal inhibitory tone. Thus, therapeutic upregulation of KCC2—either alone or in combination with receptor-based gene transfer strategies—represents a promising route for reversing central disinhibition and restoring E/I balance in chronic pain.

### Chemogenetic Modulation of DH Sensitisation

Chemogenetic technologies such as Designer Receptors Exclusively Activated by Designer Drugs (DREADDs) provide a powerful and versatile platform for next-generation pain therapies. While DREADDs have not yet reached clinical translation, they have become invaluable tools in preclinical research due to their ability to modulate neuronal activity with high temporal and cell-type precision.

DREADDs are engineered G-protein-coupled receptors that operate on a “lock-and-key” principle: each receptor variant binds exclusively to an otherwise pharmacologically inert ligand (most commonly the clozapine metabolite clozapine-N-oxide, CNO). Functionally, DREADDs are classified as excitatory (eDREADDs) or inhibitory (iDREADDs), depending on their coupling to excitatory (Gq) or inhibitory (Gi) signalling pathways, respectively. The prototypical eDREADD, hM3Dq, is a modified human M3 muscarinic receptor that, upon activation by its synthetic ligand CNO, triggers Gq-coupled signalling, mobilising intracellular calcium stores and increasing neuronal excitability. Conversely, iDREADD hM4Di (modified from M4 muscarinic receptor, hM4) activates Gi signalling, opens potassium voltage-gated channels, and suppresses neuronal firing.

In preclinical studies, DREADDs have been instrumental in identifying the functional roles of distinct DH neuron populations [227, 230] and in mapping the DH circuits underlying mechanical and thermal hypersensitivities [160]. By combining DREADD constructs with cell-type-specific promoters, their expression can be restricted to specific neuronal populations, allowing selective manipulation of excitatory or inhibitory DH neurons.

From a therapeutic perspective, iDREADD-based silencing of excitatory DH neurons (e.g., PKCγ-positive or projection neurons) could suppress pathological excitation, whereas eDREADD activation in inhibitory neurons (e.g., galanin- or parvalbumin-positive populations) could enhance inhibitory tone. Particularly promising is the chemogenetic silencing of lamina I projection neurons, which constitute a well-defined population transmitting nociceptive signals to supraspinal centres. Targeting this group with iDREADD may provide a generic and flexible strategy for treating chronic pain of various aetiologies.

### Optogenetic Modulation of DH Circuits

Over the past decade, advances in optogenetic technology have enabled selective activation/inhibition of defined DH neuron populations, significantly expanding our understanding of the circuitry organisation and nociceptive processing [231–234]. Optogenetic tools employ light-sensitive proteins, opsins, to precisely modulate neuronal excitability on a millisecond scale. Three major classes of opsins can be applied for therapeutic neuromodulation:

1. excitatory opsins (Channelrhodopsin 2), which are non-selective cation channels causing membrane depolarisation when activated by light;
2. inhibitory opsins (Halorhodopsin, Archaerhodopsin), which hyperpolarise neurons *via* activating chloride influx or proton efflux.
3. opto G protein-coupled receptor (GPCR) (opto-mu opioid receptor, opto-MOR), chimeric proteins combining the extracellular and transmembrane domains of light-sensitive rhodopsins with the intracellular (cytoplasmic) domains of the GPCR of interest.

Targeted photoactivation of excitatory opsins in inhibitory neurons can restore both phasic and tonic inhibition within the DH circuits, whereas photoactivation of inhibitory opsins in excitatory neurons can suppress their hyperexcitability. Expression of opto-MORs in excitatory neurons may decrease their activation without eliciting systemic side effects associated with conventional opioid analgesics [235].

Despite high promise, optogenetic applications pose several technical challenges. These include (1) the need for light delivery systems implantable within the limited intrathecal space, (2) light scattering across the tissue, and (3) phototoxic effects associated with prolonged exposure to blue light, which promotes activity-induced gene expression regardless of optogenetic protein expression [236]. Some of these challenges can be mitigated by using epidural fibre-optic illumination, the approach that has been successfully employed to inhibit primary afferents [237]. Switching to yellow or red light-sensitive opsins [234], or using step-function opsins that remain open after the light is switched off [238], seems to reduce phototoxicity. Light scattering can be minimised by employing infrared-activated opsins.

Effective photoactivation of DH neurons in deeper laminae II-III remains challenging, making optogenetic strategies more feasible for targeting superficial DH neurons, particularly lamina I projection neurons, which reside within ~20 μm of the spinal surface. Expression of yellow light-activated Halorhodopsin in these neurons would effectively suppress their aberrant hyperactivity in chronic pain conditions [239], while avoiding the drawbacks of blue-light stimulation.

## Gene Transfer in Pain Management: Challenges and Future Directions

Neuron-type-specific strategies for pain therapy depend on precise access to defined neuronal populations, which is currently most achievable using genetic platforms. Among the available gene approaches, viral-mediated transfer, oligonucleotide-based modulation of protein expression, and chemogenetic tools have emerged as particularly promising because they can be configured to manipulate molecular pathways within selected DH microcircuits.

Adeno-associated virus (AAV)-mediated gene transfer can provide stable, durable gene expression in the CNS, and can be adapted for conditional or cell-type-selective expression through promoter/enhancer choice and recombinase-dependent designs. Its translation, however, remains constrained by safety considerations, including potential neurotoxicity and immune responses that may reduce efficacy and, in some cases, require immunosuppression. In preclinical pain research, AAV vectors have been widely adopted, but studies still rely on broad promoters, hence non-selective targeting. A recent systematic analysis [240] identified over 40 preclinical studies employing AAV-based strategies for pain management, with none utilising neuron-type-specific promoters, and only 6 targeting synaptic transmission. At present, AAV-based approaches to modify synaptic transmission within the DH include:

1. overexpression of the GABA-synthesising enzyme GAD65 [241–243];
2. upregulation of arginine decarboxylase to elevate endogenous synthesis of NMDAR antagonist agmatine [244, 245];
3. downregulation of PICK1 to modulate AMPAR trafficking [246].

Even when AAV expression is broadly neuronal, it can lead to functionally selective outcomes depending on which cell type benefits most from the introduced approach. For example, the intrathecal delivery of vectors encoding GAD65 and the vesicular GABA transporter VGAT can broadly transduce DH neurons yet preferentially reduce pain through GAD65/VGAT expression in excitatory DH neurons, effectively shifting their neurochemical phenotype [241]. Also, vGluT2-positive terminals co-expressing GAD65/VGAT have been observed in the thalamus. This suggests a phenotypic conversion of spinothalamic projection neurons and consequent modulation of nociceptive processing at supraspinal levels.

AAV-based therapies targeting Nav1.7 (SCN9A) in primary nociceptors illustrate a complementary strategy: reducing afferent excitability to decrease nociceptive drive to the spinal DH in pain conditions [247]. This is strongly supported by human genetics, in which LoF SCN9A mutations cause congenital insensitivity to pain [248], and by experimental evidence that Nav1.7 is upregulated in multiple pain states. Two conceptually distinct genetic approaches have been explored:

- Expression of peptides that competitively disrupt the interaction between the regulatory protein (collapsin response mediator protein 2, CRMP2) and Nav1.7 to reduce Nav1.7 currents [249];
- epigenetic repression using CRISPR-based systems (KRAB-dCas9) that downregulate Nav1.7 transcription without permanently editing the genome [250].

*In vivo* repression of Nav1.7 using AAV-delivered KRAB-dCas9 produced long-lasting analgesia across inflammatory and neuropathic pain models without major impairment of sensory function [251]. Basically, Nav1.7 should no longer be viewed as an exclusively peripheral target, since Nav1.7 mRNA and protein expression have been reported in projection neurons, including NK1R-positive cells, in the human DH [252], highlighting direct Nav1.7 contribution to central hyperexcitability. Studies in rodent pain models have described an increased Nav1.7 expression in DH neurons [253], further linking Nav1.7 to DH hyperexcitability, thus the potential for targeting.

Achieving cell-type-specificity is an essential step. Conceptually, the use of viral vectors driven by cell-type-specific promoters (excitatory vs inhibitory vs projection neurons *via* vGluT2, GAD65, or Lypd1 targeting) can aim to rebalance E/I in DH microcircuits. In practice, this requires not only promoter/enhancer selection but also spatial restriction, because many genetic markers are shared across spinal and supraspinal neurons, despite strong evidence for neuron-type-specific changes in chronic pain [240, 254]. Achieving true specificity will require optimised viral designs in combination with spatially restricted delivery, since intrathecal delivery results in broad cerebrospinal fluid distribution. Indeed, intrathecal delivery—a well-established route for spinal administration to deliver viral constructs or oligonucleotides directly into the cerebrospinal fluid, bypassing the BBB—is limited by cerebrospinal fluid flow and rostral dispersion, which can cause unintended transgene expression in supraspinal regions (depending on serotype and conditions), even at low doses [255–257].

Recent advances in subpial delivery provide a potential solution by limiting rostral spread, thereby enabling more segmentally restricted DH targeting than intrathecal infusion. Precisely dosed subpial injections can restrict expression to the ipsilateral DH across a few spinal segments [258], with minimal retrograde spread to the brain [241]. Although subpial delivery is technically demanding (requiring microsurgical laminectomy), it currently represents the most precise and region-restricted method for spinal gene therapy. Its clinical use, however, may be suited for severe, treatment-resistant pain, where potential risks are justified by therapeutic benefits.

Translational gene-based pain management will depend on progress in several innovations:

- optimised vector/capsid design with enhanced spinal tropism;
- genetic control systems to constrain expression to selected cell types, ideally inducible/self-limiting expression (Cre-dependent or drug-inducible system);
- spatially precise delivery to prevent supraspinal off-target expression.

Progress on all three combinations, integrated with chemogenetic control for reversibility, could realistically bridge mechanistic insights on DH sensitisation with clinically viable, precision-targeted therapies for chronic pain.

## Concluding Remarks

Over the past decades, advances in molecular, cellular, and systems neuroscience have substantially refined current understanding of the mechanisms underlying chronic pain. Pain chronification is now widely viewed as a form of maladaptive neuroplasticity within the spinal DH, where disruption of E/I balance reshapes nociceptive processing and drives persistent pain signalling. These adaptations occur through cell-type-specific mechanisms involving excitatory and inhibitory interneurons, projection neurons, and synaptic interactions within DH microcircuits. While traditional pharmacological treatments focus on modifying peripheral nociceptor inputs or broad central pathways, their modest efficacy and frequent adverse effects highlight the need for therapies that target central mechanisms with neuron-type specificity. Emerging tools from molecular genetics, viral vector engineering, and chemogenetics increasingly make such precision feasible. By manipulating defined key players of synaptic transmission (AMPARs, GABA_A_Rs, GlyRs, and KCC2) within identified DH neuron populations, emerging strategies aim to restore E/I balance rather than suppress nociceptive outputs.

Next-generation therapies are likely to integrate gene-transfer technologies, chemogenetic or inducible control systems, and cell-type-selective promoter/enhancer elements to enable precise and potentially reversible modulation of DH excitability. Innovations in vector delivery will be equally important, including methods designed to restrict expression to the spinal cord (e.g., subpial administration) and engineering AAV capsids with improved spinal tropism and reduced off-target transduction for safe transgene expression. However, major scientific, technical, and ethical challenges still limit clinical translation. Key obstacles include achieving reliable neuron-type specificity in the human spinal cord, controlling the magnitude and duration of transgene expression, and ensuring long-term safety, particularly critical for sensory discrimination and motor function. Translation is further impeded by the absence of practical methods to directly verify spinal microcircuit engagement, as invasive electrophysiological recordings and optical readouts that validate target modulation in animal models are not readily applicable in humans. The limited availability of robust biomarkers for DH excitability and central sensitisation also complicates patient stratification, dose selection, treatment monitoring, and outcome assessment.

Moving from mechanistic insights to clinical applications will require close integration of basic pain neuroscience with molecular engineering, advanced imaging, and biomarker development, supported by carefully designed clinical trials. Once these barriers are addressed *via* multidisciplinary effort, mechanism-based, neuron-type-specific interventions could shift pain treatment from symptomatic suppression toward circuit-level restoration of normal sensory function, offering new options for otherwise treatment-resistant chronic pain syndromes.
